# Study on the Preparation and Performance of Silicone-Modified Phenolic Resin Binder for Rail Grinding Wheels

**DOI:** 10.3390/molecules28083400

**Published:** 2023-04-12

**Authors:** Pengzhan Liu, Tianshun Yuan, Jin Peng, Wenjun Zou, Furen Xiao

**Affiliations:** 1 Henan Engineering Lab for Super-Hard Grinding Composites, College of Materials Science & Engineering, Henan University of Technology, Zhengzhou 450007, China; 2 Key Lab of Metastable Materials Science & Technology, Hebei Key Lab for Optimizing Metal Product Technology and Performance, College of Materials Science & Engineering, Yanshan University, Qinhuangdao 066004, China

**Keywords:** silicone-modified, phenolic resin, rail grinding wheel, methyl-trimethoxy-silane, preparation, resin performance

## Abstract

A scheme for manufacturing heavy-duty rail grinding wheels with silicone-modified phenolic resin (SMPR) as a binder in the field of rail grinding is presented to improve the performance of grinding wheels. To optimize the heat resistance and mechanical performance of rail grinding wheels, an SMPR for industrial production of rail grinding wheels was prepared in a two-step reaction using methyl-trimethoxy-silane (MTMS) as the organosilicon modifier by guiding the occurrence of the transesterification and addition polymerization reactions. The effect of MTMS concentration on the performance of silicone-modified phenolic resin for application in rail grinding wheels was investigated. The molecular structure, thermal stability, bending strength, and impact strength values of the SMPR were characterized by Fourier transform infrared spectroscopy (FTIR), thermogravimetric analysis (TGA), and mechanical property testing, and the effect of MTMS content on the resin properties was investigated. The results indicated that MTMS successfully improved the performance of the phenolic resin. The thermogravimetric weight loss temperature of the SMPR modified by MTMS with 40% phenol mass at 30% weight loss is 66% higher than that of common phenolic resin (UMPR), exhibiting the best thermal stability; in addition, its bending strength and impact strength were enhanced by approximately 14% and 6%, respectively, compared with those of common UMPR. This study utilized an innovative Bronsted acid as a catalyst and simplified several intermediate reactions in the conventional silicone-modified phenolic resin technology. This new investigation of the synthesis process decreases the manufacturing cost of the SMPR, liberates it from the restrictions of grinding applications, and enables the SMPR to maximize its performance in the rail grinding industry. This study serves as a reference for future work on resin binders for grinding wheels and the development of rail grinding wheel manufacturing technology.

## 1. Introduction

Surface defects on in-service rails are inevitable after prolonged use, reducing their service life and the safety of train operation [1,2]. Currently, rail grinding is the primary technological procedure utilized to repair rail surface problems [3,4]. Heavy-duty grinding wheels made of phenolic resin binder are frequently required to execute grinding operations on rails in the rail grinding process [5,6]. However, grinding wheels are typically required to grind rails at high grinding pressures, and environmental protection concerns prohibit the use of coolants during rail grinding [7,8]. In consequence, rail grinding wheels are frequently subjected to extreme grinding squeezing, grinding impact behavior, and high grinding temperatures when grinding rails [9,10]. In light of the aforementioned considerations, the phenolic resin binder for preparing rail grinding wheels has to possess excellent mechanical and thermal stability to ensure the performance of the rail grinding wheels.

However, the phenolic hydroxyl and methylene in the structure of conventional phenolic resin are extremely vulnerable to oxidation, resulting in the poor heat resistance of rail grinding wheels constructed with conventional phenolic resins [11,12]. In addition, the rigid benzene rings in the molecular chain structure of the conventional phenolic resin used to manufacture rail grinding wheels are only connected by methylene groups after curing, which makes the mechanical properties of the conventional phenolic resin relatively average and also leads to the poor mechanical performance of rail grinding wheels [13,14]. The mediocre thermal stability and mechanical performance of phenolic resins will reduce the service life and grinding ability of rail grinding wheels, thereby impeding the advancement of rail grinding technology. Therefore, it is the development trend of the rail grinding wheel manufacturing industry to modify the special phenolic resin binder for rail grinding wheels to improve the heat resistance and mechanical strength of the grinding wheels.

Organosilicon compounds are a category of polymer material modifiers with excellent thermal stability, low-temperature toughness, and water resistance that can be used to chemically modify the thermal stability and mechanical properties of phenolic resins. Numerous pieces of research have demonstrated the efficacy of the silicon modification method [15,16]. Cheng et al. [17] developed a siloxane modifier (4,4’-(1,5-dipropyl-3, 3-diphenyl-1,1,5,5-tetramethyltrisiloxane) bis-2-methoxyphenol) to improve the brittleness and thermal stability of phenolic resins. The results of the study showed that at a high conversion range, the silicone-modified phenolic resins (SMPR)/hexamethylenetetramine(HMTA) exhibits lower activation energy than UMPR/HMTA, indicating a stronger cure reaction activity for the former. Du et al. [18] conducted theoretical and experimental investigations into the pyrolysis mechanisms of silicone-modified phenolic resin under high temperatures. This work elucidated the fundamental pyrolysis and carbonization mechanism of silicone-modified phenolic resins by combining reactive force field MD simulations with experimental characterization and discussed the effect of the introduction of silicone compounds on the thermal stability and high-temperature residual weight of the modified phenolic resins. Yin et al. [19] demonstrated the synthesis of low-density and thermally stable phenolic resin/silicone (UMPR-Si) hybrid aerogels using phenolic resin (UMPR), methyl-trimethoxy-silane (MTMS), hexamethylenetramine (HMTA) and ethylene glycol (EG) solution. This study revealed that the silicone-modified phenolic resin can have better thermal stability and mechanical properties.

Organosiloxanes have Si-O bonds (460 kJ/mol), which have greater bonding energy than C-O bonds (326 kJ/mol). The introduction of Si-O bonds into the molecular structure of phenolic resins to partially block the phenolic hydroxyl groups is an effective way to improve the heat resistance properties of phenolic resins [17]. However, the current general method of modifying phenolic resins with organosilicon compounds is to first hydrolyze the organosilicon under specific conditions to form oligomers containing Si-O-Si and Si-OH structures in the molecular structure and then react the hydrolysis products with the phenolic hydroxyl groups in the phenolic resin to prepare the silicone phenolic resin. Not only is the reaction course of this process complex and inefficient, with multiple elementary reactions such as hydrolysis and self-polymerization of siloxane monomer, phenolic self-polymerization, and copolymerization of siloxane monomer and phenolic aldehyde, but it is also difficult to control the degree of reaction polymerization. The conventional modification method is, therefore, unsuitable for the industrial manufacturing of resin binders for rail grinding wheels.

To simplify the tedious process of organosilicon-modified phenolic resin and improve the product stability of the modified phenolic resin, MTMS (Methyl-trimethoxy-silane) was selected as the organosilicon modifier in this study. It was directly transesterified with phenol under acidic conditions to obtain the methyl-phenoxy-silane intermediate, followed by further reaction with paraformaldehyde (POM). The silicone-modified phenolic resin (SMPR) for rail grinding wheels was prepared by a convenient and low-cost novel synthesis method. Notably, this reaction process avoided multiple intermediate reactions (such as hydrolytic self-polymerization of organic silicon monomer, polymerization of phenol and formaldehyde, copolymerization of organic silicon monomer and phenolic, etc.) in the conventional modification process and utilized a newly developed Bronsted acid as an acidic catalyst, which has never been used in industrial production before. Silicone phenolic resin (SMPR) with great heat resistance and considerable mechanical strength was synthesized in only two steps by submitting silicone to transesterification reactions and polymerization reactions with phenol. Furthermore, FTIR, DSC, SEM, and TGA testing methods were implemented for structural characterization and performance analysis of SMPR with varying MTMS content to investigate the effect of MTMS dosage on the performance of the special SMPR for rail grinding wheels, and its mechanism was discussed. The results of this study provide references for the development of high-performance rail grinding wheels and phenolic binders for abrasive grinding tools.

## 2. Results and Discussion

### 2.1. Synthesis Theory and Infrared Spectral Analysis of the Silicone-Modified Phenolic Resin

**Figure 1:**
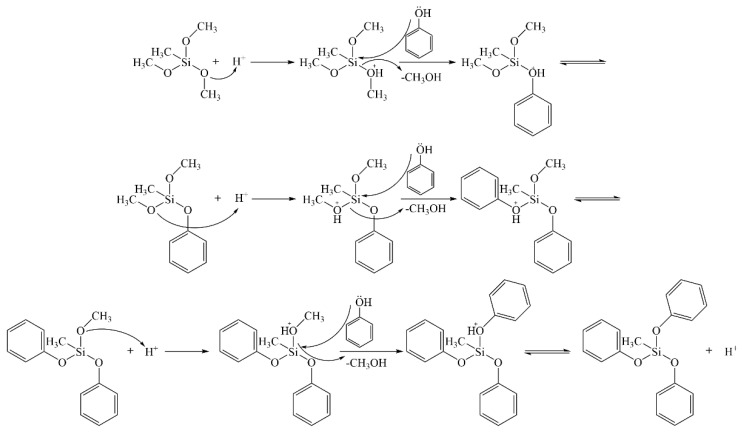
Preparation of methyl-trimethoxy-silane intermediates by transesterification.

The synthesis process of silicone-modified phenolic resin is mostly composed of two steps. Firstly, phenol and MTMS undergo transesterification reactions catalyzed by TsOH to generate methyl-trimethoxy-silane intermediates. In an acidic environment, phenol will replace the methoxy group in MTMS to form the methyl-trimethoxy-silane intermediate. As a result of the weak bonding energy of the hydrogen-oxygen bond (O-H) in the phenol structure, hydrogen ions (H+) are liberated during this reaction to maintain the reaction system in the acidic environment. This causes the continual interaction of phenol with the unreacted methoxy group in MTMS to produce monosubstituted, disubstituted, and trisubstituted methyl-trimethoxy-silane intermediates. This procedure is illustrated in Figure 1.

**Figure 2:**
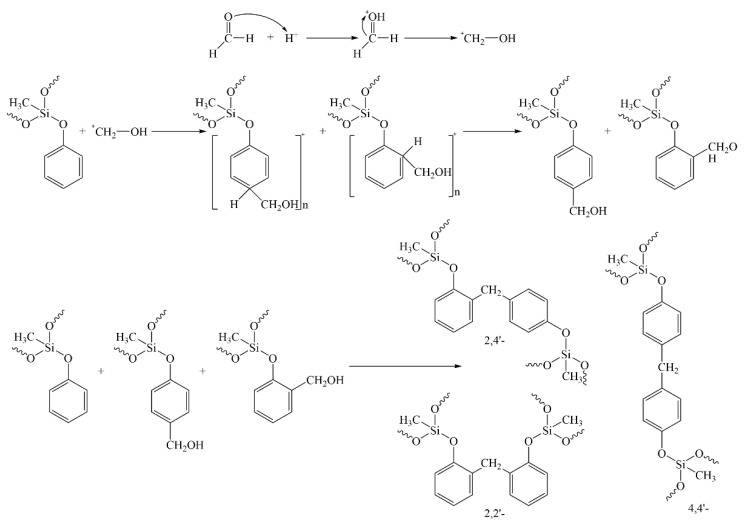
Reaction of methyl-trimethoxy-silane intermediates with POM.

Secondly, paraformaldehyde (POM) is added to the reaction system to further react with the generated methyl-trimethoxy-silane intermediates. In an acidic environment, POM will depolymerize and first react with the ortho- and para-positions of the benzene ring in methyl-trimethoxy-silane intermediates to produce two transition state intermediates. These two intermediates then continue to react with methyl-trimethoxy-silane to form three oligomers with the formulas 2,2’, 2,4’, and 4,4’. As the degree of polymerization reaction increases, the silicone-modified phenolic resin (SMPR) with a particular degree of cross-linking in the molecular structure is finally generated. This process is illustrated in Figure 2.

**Figure 3:**
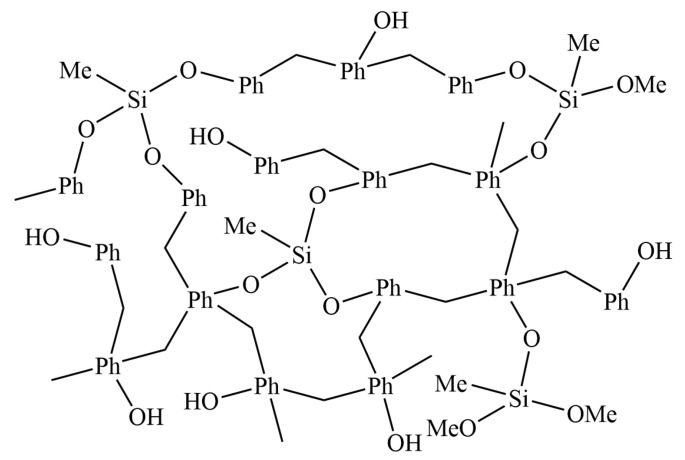
Structure schematic of SMPR.

The schematic molecular structure of the synthesized SMPR is shown in Figure 3. The Me and Ph indicate methyl and benzene rings, respectively, in the diagram. Notably, MTMS is particularly vulnerable to hydrolysis in the presence of water in an acidic environment; hence, the acid catalyst chosen must be nearly water-free. As an acid catalyst, virtually anhydrous p-toluenesulfonic acid (TsOH) was utilized so that phenol and MTMS would undergo transesterification rather than hydrolytic condensation polymerization. The selection of TsOH as the acid catalyst was the result of lengthy experimentation, and it had never been utilized in industrial production before. This is one of the significant innovations in this work.

**Figure 4:**
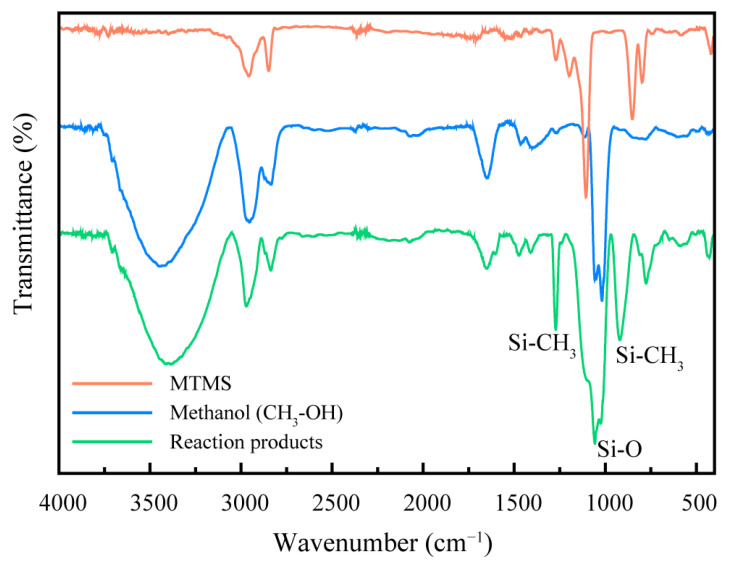
Infrared spectra of methyl-trimethoxy-silane (MTMS), methanol and reaction products of MTMS with phenol.

To confirm the transesterification reaction between MTMS and phenol, the infrared spectroscopy of the low boiling point products produced in the reflux phase in the first step was analyzed and compared with the infrared spectra of analytical purity methanol and methyl-trimethoxy-silane. The results are shown in Figure 4. Compared to methanol and MTMS, the figure illustrates that the reflux product exhibited three absorption peaks at 1271 cm^−1^, 920 cm^−1^, and 775 cm^−1^ that did not appear in methanol and MTMS, including the bending vibration peak of Si-CH_3_ at 1271 cm^−1^ and 775 cm^−1^ and the stretching vibration peak of the Si-O bond at 920 cm^−1^. In addition, the IR spectrum indicated that 3428 cm^−1^ is the stretching vibration peak of hydroxyl (-OH), 2963 cm^−1^ and 2836 cm^−1^ are the stretching vibration peaks of the C-H bond, 1408 cm^−1^ is the bending vibration peak of hydroxyl (-OH), 1469 cm^−1^ and 1038 cm^−1^ are the bending vibration peaks of the C-H bond.

The infrared spectrum analysis revealed the presence of methanol, a by-product of the transesterification reaction between MTMS and phenol, and unreacted MTMS in the low boiling point products generated during the reflux stage. According to the mechanism of transesterification for the synthesis of methyl-trimethoxy-silane intermediates, the production of methanol indicates the occurrence of the transesterification reaction and the formation of methylphenoxyilane intermediates.

**Figure 5:**
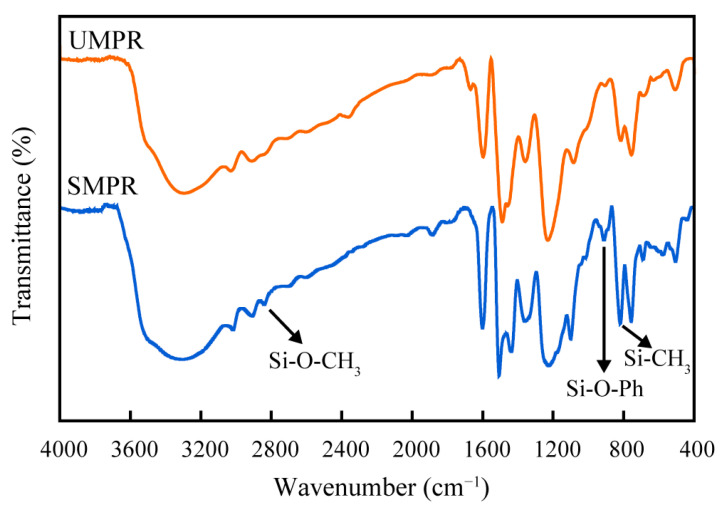
Infrared spectra of unmodified phenolic resin and silicone-modified phenolic resin.

Figure 5 displays the results of infrared spectroscopy tests conducted on silicone-modified phenolic resins with MTMS content of 0 (i.e., phenolic resin without MTMS modification UMPR) and with MTMS content of 40% phenol mass (SMPR). In the UMPR infrared spectrum test results, a stretching vibration peak of hydroxyl emerged at 3296 cm^−1^, a stretching vibration peak of the C-H bond appeared at 3030 cm^−1^ and 2911 cm^−1^, and a stretching vibration absorption peak of the benzene ring skeleton appeared at 1597 cm^−1^ and 1441 cm^−1^. In the SMPR infrared spectrum test results, the stretching vibration peak of hydroxyl appeared at 3299 cm^−1^, the stretching vibration peak of the C-H bond appeared at 3017 cm^−1^ and 2909 cm^−1^, and the stretching vibration absorption peak of the benzene ring skeleton appeared at 1601 cm^−1^ and 1550 cm^−1^. Moreover, the stretching vibration peaks of C-O bonds between the benzene ring and phenolic hydroxyl groups were detected at 1231 cm^−1^ and 1226 cm^−1^ for UMPR and SMPR, respectively. There is no discernible distinction between UMPR and SMPR, indicating that the two phenolic resins have structural similarities [20]. Compared with the unmodified phenolic resin, the MTMS-modified silicone phenolic resin exhibited stretching vibration peaks of Si-O-CH_3_ at 2840 cm^−1^ and 1101 cm^−1^, stretching vibration peaks of Si-O-Ph at 912 cm^−1^, and bending vibration peaks of Si-CH_3_ at 819 cm^−1^. The bending vibration peak of Si-CH_3_ at 819 cm^−1^ overlapped with the characteristic peak of para substitution of the benzene ring, increasing the intensity of the absorption peak, but the characteristic absorption peak of Si-O-Si did not appear in the range from 1030 cm^−1^ to 1078 cm^−1^ [21]. This demonstrates that the Si-C and Si-O bonds in the organosilicon compound MTMS were successfully incorporated into the macromolecular chain structure of the modified phenolic resin and that the silicone-modified phenolic resin (SMPR) was successfully synthesized, thereby altering the heat resistance and mechanical properties of the conventional phenolic resin [16,22].

### 2.2. Effect of MTMS Content on Fluidity of the Silicone-Modified Phenolic Resin

**Figure 6:**
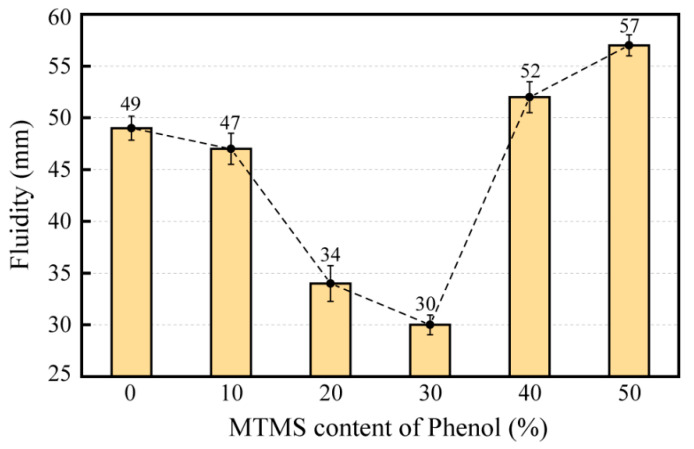
Fluidity of SMPR with different MTMS content of phenol.

Fluidity is a measurement of the flow of the resin system, which has a direct effect on the performance of the grinding wheel resin bond during the grinding wheel manufacturing process. Because the resin bond required to produce the grinding wheel cannot be reworked once it has been formed with the abrasives, the fluidity of the resin impacts its machinability in the grinding wheel production process [23]. Excessive resin fluidity will result in material overflow during the preparation of rail grinding wheels, which reduces the adhesion of the resin to abrasive and consequently leads to a decline in grinding wheel performance; low resin fluidity will cause higher forming pressure and temperature in the preparation of grinding wheels, which will cause many negative effects, such as the unstable quality of grinding wheels [24]. Figure 6 depicts the fluidity test results for SMPR with different MTMS contents.

Figure 6 illustrates that with the increase in MTMS content, the fluidity of resin tended to decrease first and then increase. The highest SMPR fluidity was 57 mm at 50% MTMS content, whereas the lowest SMPR fluidity was 30 mm at 30% MTMS content. This is attributed to the fact that the introduction of MTMS blocked a portion of the phenolic hydroxyl groups, hence increasing the local cross-linked structure in the resin structure. It caused the cross-linking density of the resin to rise, thus reducing the fluidity of the resin. When the MTMS content exceeded 30%, the likelihood of o-Methoxyaniline (-OCH_3_) in the MTMS structure fully participating in the transesterification reaction decreased as the MTMS content increased. This led to a reduction in the relative amount of three-dimensional cross-linked structures formed by the dealcoholization reaction between MTMS and hydroxyl (-OH) in phenol, resulting in a decrease in the cross-link density of the resin, which caused an increase in the fluidity of the resin [25]. Overall, although the fluidity of the six groups of resin specimens satisfied the requirements for the manufacture of rail grinding wheels, the phenolic resins prepared when the MTMS dosage is 0%, 10%, 40%, and 50% of the phenol mass are more suitable for the production and molding process of rail grinding wheels.

### 2.3. Effect of MTMS Content on Curing Temperature of the Silicone-Modified Phenolic Resin

**Figure 7:**
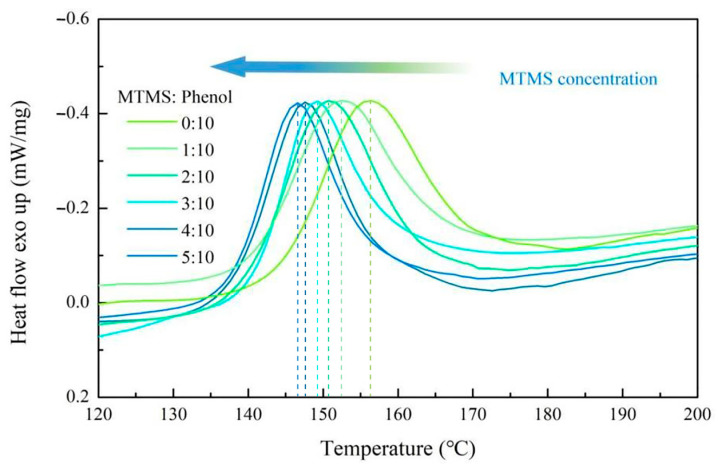
DSC of SMPR with different MTMS content of phenol.

The production cost of grinding wheels is strongly affected by the curing temperature of the resin binder. Lower curing temperatures are preferred because they reduce energy use and costs. The silicone-modified phenolic resin and the curing agent hexamethylenetetramine (HMTA) were capable of undergoing a cross-linking cure reaction within a specific temperature range to form an insoluble three-dimensional network structure. DSC can be utilized to measure the temperature required for the cross-linking reaction to occur, which is the curing temperature of the resin binder for the preparation of rail grinding wheels. The temperature at which phenolic resins cross-link and cure are typically between 100 °C and 200 °C. Figure 7 depicts the DSC curves of SMPR with different MTMS contents under a nitrogen environment at a heating rate of 10 °C/min from 120 °C to 200 °C, which are the curing temperature curves of SMPR with various MTMS contents.

As shown in Figure 7, the peak curing temperatures of SMPR with various MTMS contents ranged from 140 to 160 °C, and the peak curing temperatures of SMPR decreased as the MTMS content increased. The lowest curing temperature of SMPR was 147 °C when the MTMS content was 50% of phenol. This temperature is 9 °C lower than the peak curing temperature (156 °C) of UMPR. Therefore, SMPR has a lower curing temperature compared to UMPR. This makes SMPR have a higher practical value in the grinding wheel manufacturing industry. From the molecular chain structure of SMPR, it is evident that an increase in MTMS content increases the degree of SMPRs molecular chain branching, resulting in a significant improvement in its tendency to cross-link and cure. In addition, the incorporation of MTMS indirectly reduces the density of benzene rings in the SMPR structure, thereby decreasing the steric hindrance to the molecular chain movement of the resin. With an increase in MTMS content, the combined effect of these factors caused the peak curing temperature of SMPR to shift toward the low-temperature region.

### 2.4. Effect of MTMS Content on Thermal Properties of the Silicone-Modified Phenolic Resin

**Figure 8:**
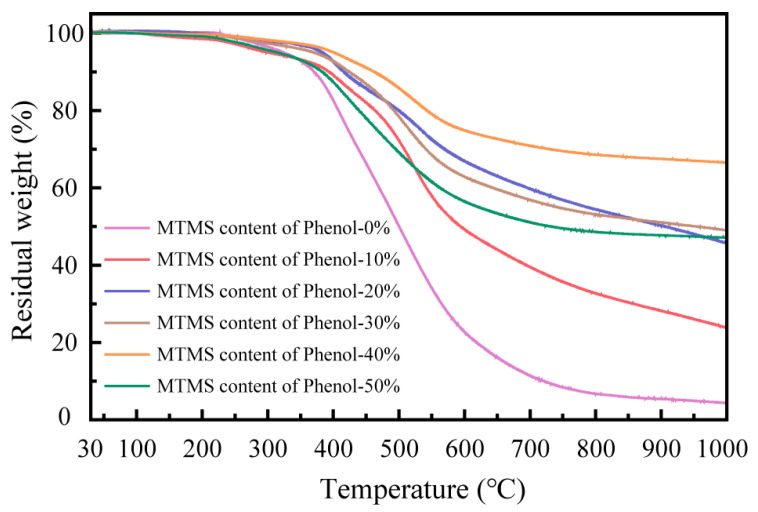
TGA of phenolic resin modified with different content of MTMS at 30~1000 °C.

**Figure 9:**
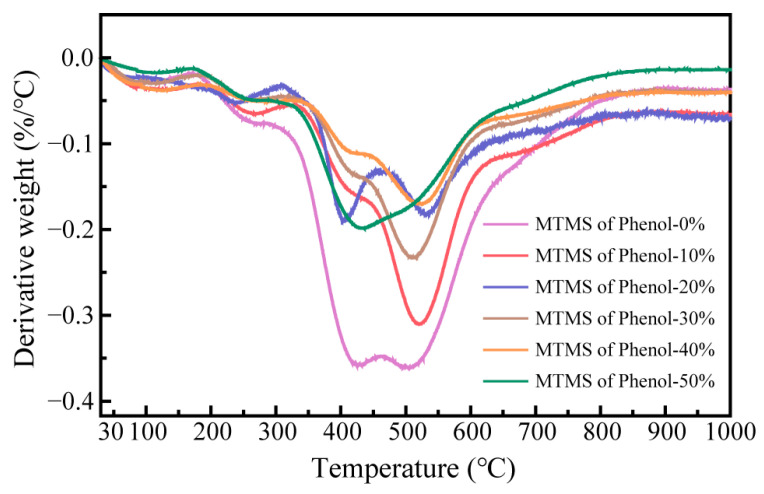
DTG of phenolic resin modified with different content of MTMS at 30~1000 °C.

The primary objective of modifying phenolic resin with MTMS is to enhance the thermal stability of the resin in order to optimize the heat resistance of rail grinding wheels. The residual weight ratio of SMPR with different MTMS dosages was tested by thermogravimetric analysis (TGA) in the temperature range of 30~1000 °C, and the first-order differential curves (DTG) of their thermal weight loss were demonstrated. The test results are shown in Figure 8 and Figure 9.

The change in the residual weight ratio of phenolic resin between 30 °C and 1000 °C reflects its thermal weight loss. At a certain temperature, the higher the residual weight rate of the resin, the greater its thermal stability, and vice versa. As the temperature rises, the faster the rate of decrease in the residual weight rate of the resin, the higher the thermal weight loss rate of the resin, indicating that the thermal stability of the resin is poor; conversely, as the temperature rises, the slower the rate of decrease in the residual weight rate of the resin, the lower the thermal weight loss rate of the resin, indicating that the thermal stability of the resin is better [26]. The change in resin weight (TGA) and the rate of change in resin weight (DTG) demonstrate that as the temperature exceeds 300 °C, the residual weight rate of SMPR begins to fall dramatically, and the rate of resin weight loss raises with the increase in temperature, i.e., the thermal weight loss of SMPR begins to grow significantly. This implies that SMPR starts to decompose and release tiny molecule-free substances, such as free phenols and aldehydes, etc. As the temperature continues to rise, the thermal weight loss of SMPR subsequently increases, and methane, free phenols, water, and carbon dioxide may be released. Eventually, when the temperature exceeds 600 °C, the residual weight of the phenolic resin begins to decline at a slower rate, indicating that the phenolic resin starts to carbonize and release water, carbon dioxide, phenols, and benzene again with the continued increase in temperature [27,28]. In addition, when the MTMS dosage was 0%, the residual weight rate of the phenolic resin decreased the fastest with the increase in temperature and was the lowest at 1000 °C. This shows that the thermal stability of conventional phenolic resin (UMPR) before modification is considerably inferior to that of SMPR modified with MTMS. When the amount of MTMS is at 40%, the residual weight rate of the phenolic resin decreases the slowest as the temperature rises, and the residual weight rate at 1000 °C is the highest. Therefore, the SMPR prepared with 40% phenol mass of MTMS possesses excellent thermal stability.

**Figure 10:**
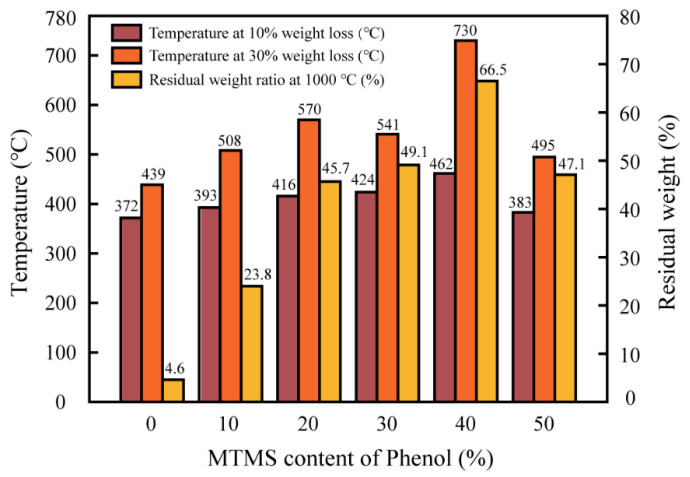
Temperature of phenolic resin modified with different content of MTMS at 10% and 30% thermal weight loss and residual weight ratio at 1000 °C.

In addition to evaluating the residual weight rate at 30~1000 °C for various MTMS dosages of SMPR, the thermal weight loss temperature at 10% weight loss and 30% weight loss of phenolic resin and the residual weight rate at 1000 °C were statistically analyzed, and the results are shown in Figure 10. In the grinding wheel manufacturing industry, the thermal weight loss temperature at 10% weight loss of resin binder can represent the initial decomposition temperature of the grinding wheel; the thermal weight loss temperature at 30% loss of resin binder can represent the maximum safe temperature that the grinding wheel can withstand during the grinding process, above which the grinding wheel risks breaking or faster wear.

Figure 10 reveals that the thermal weight loss temperature of modified phenolic resin with 40% MTMS content is approximately 360 °C higher than that of conventional phenolic resin (UMPR) at 30% weight loss and nearly 66% higher than that of UMPR; the thermal weight loss temperature of modified phenolic resin at 10% weight loss is almost 90 °C higher than the UMPR, which is roughly 24% higher than that of UMPR. Additionally, the residual weight rate of 40% MTMS-modified phenolic resin at 1000 °C is as high as 66.5%, compared to 4.6% for UMPR at the same temperature. Additionally, the modified phenolic resins with an MTMS content of 10%, 20%, 30%, and 50% phenol exhibit weaker thermal stability than the modified phenolic resin with an MTMS content of 40% phenol because of differences in cross-link density and the degree of blockage of phenolic hydroxyl groups. The improvement of thermal stability of phenolic resin modified by MTMS is primarily attributable to the fact that MTMS blocks a portion of the phenolic hydroxyl group (-OH) during the synthesis of phenolic resin and introduces silicon-oxygen bonds (Si-O) with bond energies much greater than those of carbon (C-C) in the phenolic resin, resulting in the formation of macromolecular phenolic resin structures with high cross-linking density. Hence improving the thermal stability of the resin. The degree of polymerization of polymers also affects their thermal stability [29,30]. The effect of polymerization degree on the thermal stability of SMPR remains to be further explored [31]. In addition to the gain in thermal stability, the performance advantage of SMPR is also attributable to its high residual weight rate. Even if thermal weight loss occurs at higher grinding temperatures, the higher residual mass of SMPR as the resin binder for rail grinding wheels will minimize the impact on the mechanical properties and grinding performance of the grinding wheels due to the decrease in density of grinding wheels caused by the thermal decomposition of the binder.

### 2.5. Effect of MTMS Content on Mechanical Properties of the Silicone-Modified Phenolic Resin

**Figure 11:**
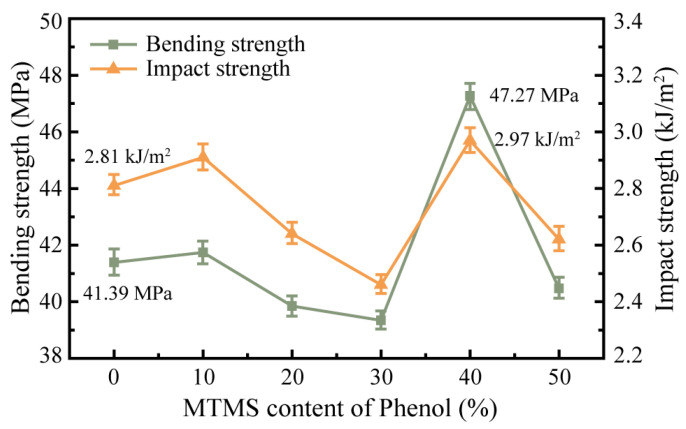
Bending strength and impact strength of phenolic resin modified with different content of MTMS.

The bending strength and impact strength of the resin binder will have a substantial effect on the bending and impact performances of the grinding wheels; therefore, the mechanical strength of the modified phenolic resin dictates the mechanical properties of the rail grinding wheel. The bending and impact strengths of SMPR with various MTMS doses were tested, and the results are depicted in Figure 11.

According to the findings of the tests, the bending strength and impact strength of the modified phenolic resin exhibited a trend of first increasing, then decreasing, then increasing, and finally decreasing as the amount of MTMS was increased. Without MTMS, the mean bending and impact strengths of unmodified phenolic resins were 41.39 MPa and 2.81 J/m^2^, respectively. The mean bending and impact strengths of modified phenolic resins increased to 41.74 MPa and 2.91 J/m^2^ when the amount of MTMS was increased to 10% by the mass of phenol. When the amount of MTMS was increased to 20% and 30% by mass of phenol, however, the bending and impact strengths of the modified phenolic resin dropped. When the amount of MTMS was 20% by mass of phenol, the average bending strength and impact strength of the modified phenolic resin was 39.85 MPa and 2.64 J/m^2^, respectively; when the amount of MTMS was 30% by mass of phenol, the average bending strength and impact strength were 39.34 MPa and 2.46 J/m^2^, respectively. Their mechanical strengths were even lower than those of unmodified phenolic resins. As the amount of MTMS was increased to 40% of the phenol mass, the bending strength and impact strength of the silicone-modified phenolic resin improved once again, with mean values of 47.27 MPa and 2.97 J/m^2^, which were 5.88 MPa and 0.16 kJ/m^2^ greater than those of the unmodified phenolic resin. At this point, the modified phenolic resin achieved the highest mechanical strength of all experimental groups, improving by approximately 14% and 6%, respectively, over the unmodified phenolic resin. The bending and impact strengths of the modified phenolic resin declined to mean values of 40.47 MPa and 2.62 J/m^2^, respectively, when the amount of MTMS was increased to 50% of the phenol mass.

The variation in cross-linking density of the modified phenolic resin is responsible for the change in mechanical strength with varying MTMS dosage [32]. MTMS serves as the network node for the cross-linked network of SMPR [33]. Little amounts of MTMS introduced during the synthesis of phenolic resins can increase the cross-linking sites in the structure of the phenolic resin, hence enhancing the cross-linking density of the phenolic resin. Therefore, when the amount of MTMS was increased from 0% to 10%, there was a tendency for the mechanical strength of SMPR to increase. Nevertheless, when the amount of MTMS was increased from 10% to 20% and 30%, the cross-linking sites in the molecular structure of phenolic resin increased excessively, impeding the movement of the chain segments in the molecular structure of phenolic resin. This increased the site resistance of the reaction between the modified phenolic resin and urotropine, preventing the modified phenolic resin from curing adequately. Thus, the macroscopic manifestation of the reduction in the mechanical strength of SMPR. The methoxy group (CH_3_O-) may not fully participate in the transesterification reaction during the synthesis of the modified phenolic resin when the amount of MTMS is increased to 40%. This resulted in a relative decrease in the cross-link density of the modified phenolic resin and a reduction in the difficulty of polymer chain segment movement in the phenolic resin, allowing the modified phenolic resin to fully cross-link and cure with urotropine. Additionally, therefore, the bending and impact strengths of the modified phenolic resin demonstrate an increase and are greater than those of the modified phenolic resin containing 10% MTMS. Eventually, with the excessive addition of MTMS, the utilization of MTMS in the synthesis reaction dropped dramatically when the addition of MTMS was increased to 50%, resulting in the presence of a large number of reaction by-products and redundant small molecules in the SMPR generated by the process. This affected the macroscopic mechanical property performance of the modified phenolic resin, leading to a decline in the bending and impact strengths again.

**Figure 12:**
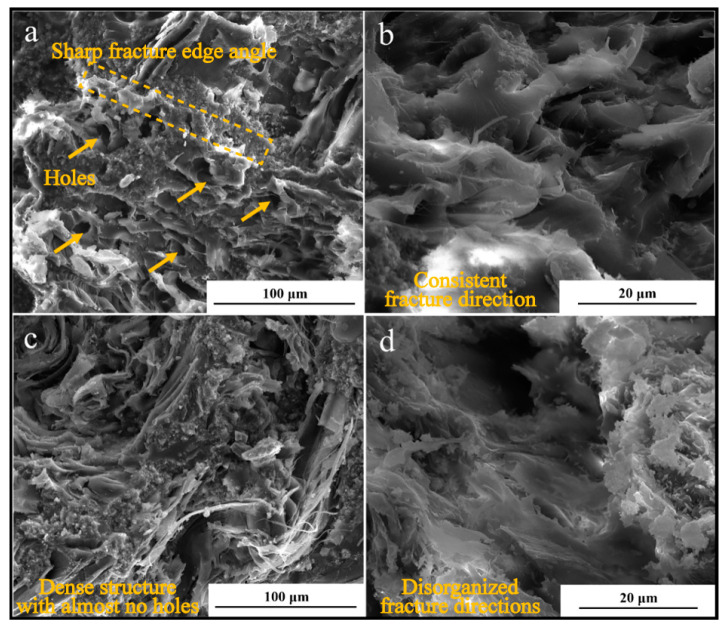
Section morphology: (**a**) UMPR section morphology at 1200×; (**b**) UMPR section morphology at 5000×; (**c**) 40% MTMS modified SMPR at 1200×; (**d**) 40% MTMS-modified SMPR at 5000×.

Figure 12 depicts the section morphologies of MTMS-modified SMPR with 40% phenol content and unmodified conventional UMPR, which were observed after impact performance testing in order to further investigate the essence of the excellent mechanical strength of SMPR relative to UMPR. Before SEM observation, a thin layer of metallic platinum was sprayed onto the surface of SMPR and UMPR specimens using an ion sputterer. Figure 12a,b exhibit the impact cross-sections of conventional UMPR under 1200 magnification (1200×) and 5000 magnification (5000×), whereas Figure 12c,d exhibit the impact cross-sections of SMPR modified with 40% MTMS under 1200 magnification (1200×) and 5000 magnification (5000×). Figure 12 demonstrates that the fracture surface structures of SMPR and UMPR are different. Many holes and sharp fractures appear on the impact fracture surface of UMPR, and the fracture happens in the same direction, which is characteristic of a brittle fracture. While the impact fracture surface of SMPR is free of holes and has high densities, the fracture direction tends to be dispersed, indicating a transition from brittle to ductile fracture.

These phenomena demonstrate that the transesterification reaction between some phenolic hydroxyl group (-OH) in the SMPR structure and the methoxy group (-OCH_3_) in the MTMS decreases the amount of free phenol and free aldehyde in the resin, thereby reducing the release of small molecules such as free phenol and aldehyde from SMPR during curing. Hence, the interior of SMPR is macroscopically devoid of pores and has a high density. In addition, studies have shown that the insertion of organic silicon into the structure of phenolic resin through chemical modification can also reduce the interfacial energy on the surface of the resin powder particles and enhance their compatibility with dispersion [34]. This boosts the adhesive abilities and mechanical properties of the resin. The primary factor influencing the mechanical qualities of grinding wheel products is the binding force between resin and filler [35]. Many holes on the fracture surface of UMPR will degrade the bond between the resin and the filler [36]. This explains why UMPR has poorer mechanical performance. In contrast, there are few pore structures in SMPR, which allows the filler to be tightly bonded during the resin fracture process. As a result, the filler can adhere firmly to the resin and absorb a significant amount of energy during the deformation and fracture processes, resulting in a significant improvement in the mechanical properties of SMPR.

### 2.6. Grinding Tests on Rail Grinding Wheels Prepared with Silicone-Modified Phenolic Resins with Different MTMS Contents

**Figure 13:**
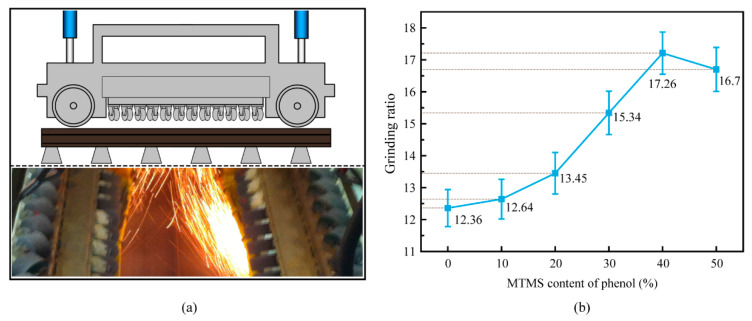
(**a**) The structure and grinding process of the HSG rail grinding mechanism; (**b**) the grinding ratio of the rail grinding wheels manufactured with silicone-modified phenolic resins containing different MTMS.

The fundamental purpose of preparing MTMS-modified silicone phenolic resin is to enhance the heat resistance and strength of the phenolic resin bond in order to improve the durability (i.e., grinding life) of rail grinding wheels. The grinding ratio is one of the key indicators to evaluate the strength and durability of grinding wheels during the grinding process. The grinding ratio is the grinding weight loss ratio between the rail and the grinding wheel, and the higher the grinding ratio indicates the better the durability of the grinding wheel [37]. In this study, grinding tests were conducted on rail grinding wheels prepared with different MTMS contents of silicone-modified phenolic resin on steel rails using the “HSG” high-speed grinding train, and the grinding ratios were calculated to reflect the effects of various MTMS contents of silicone-modified phenolic resins on the performance of rail grinding wheels. All rail grinding wheels in the grinding tests were of the same formulation, differing only in the type of resin bond. In the grinding unit of the HSG grinding mechanism, 24 grinding wheels were used. The test grinding settings were the HSG train’s set parameters as follows: the speed of the grinding train was 60 km/h, and the grinding load was 3000 N. Each set of grinding tests was conducted three times over a distance of 3 km. The detailed process was described in full in a prior work [6]. In addition, future research will focus on the effects of diverse silicone-modified phenolic resin binders on the performance of rail grinding wheels. The structure and grinding process of the HSG rail grinding mechanism are shown in Figure 13a, and the grinding ratio of the rail grinding wheels manufactured with silicone-modified phenolic resins containing different MTMS contents are shown in Figure 13b.

Figure 13b demonstrates that the grinding ratio of the rail grinding wheels increased and then decreased as the MTMS content in the silicone phenolic resin binder used to manufacture the wheels increased, given the same grinding wheel manufacturing process, formulation, and grinding parameters. The grinding ratio of the rail grinding wheel prepared with unmodified phenolic resin (UMPR) is clearly the lowest, indicating that the service life of the rail grinding wheel prepared with unmodified phenolic resin as the binder is the shortest, whereas the grinding ratio of the rail grinding wheels prepared with 40% MTMS-modified silicone phenolic resin (SMPR) is the highest, approximately 39.6% higher than that of the rail grinding wheel prepared with unmodified phenolic resin (UMPR). Results demonstrated that the rail grinding wheel with a 40% MTMS modified-silicone phenolic resin bond has the longest service life and is the most durable. As well, the grinding ratio of the rail grinding wheels rose as the amount of MTMS utilized to modify silicone phenolic resin increased. This is because the heat stability of silicone-modified phenolic resin increases as the amount of MTMS increases, and the resin’s mechanical properties also alter slightly. Grinding heat is the primary contributor to the quick wear of resin-based rail grinding wheels [38,39]. The improvement in the thermal stability of the resin bond and the rise in its residual weight after grinding heat loss can significantly contribute to the extension of the service life of rail grinding wheels, thereby enhancing the economic benefits of rail grinding maintenance. The grinding ratio of rail grinding wheels decreased, however, when the MTMS dosage was 50 percent phenol. This is consistent with the decreased thermal stability and mechanical properties of 50% MTMS-modified phenolic resin.

## 3. Experiment

### 3.1. Preparation of Silicone-Modified Phenolic Resin

First, the phenol and MTMS were homogenized mechanically at room temperature, and then p-toluenesulfonic acid (TsOH) was added. Afterward, the temperature was raised to 160 °C, and the reaction was held under continuous stirring and reflux until the end of the reaction while protected by an N_2_ atmosphere. The reaction was then cooled to 90 °C and POM was added, followed by a slow ramp-up in temperature to 110 °C to continue the reaction. When the reactants become gelatinous, the reaction should be terminated, and the pH should be adjusted to neutral.

Second, the reaction product was placed directly in a vacuum-drying oven at 120 °C for three hours to dehydrate. Additionally, after drying, silicone-modified phenolic resin (SMPR) powder for the manufacturing of rail grinding wheels was obtained by adding hexamethylenetetramine (HMTA) with a content of 10% of the reaction product and crushing it with the reaction product to form a powder.

In this work, six groups of phenolic resin containing various amounts of MTMS were prepared, and their performance was compared. The percentage content of MTMS in the text refers to the percentage of phenol content. The specific formulas are shown in Table 1.

**Figure 14:**
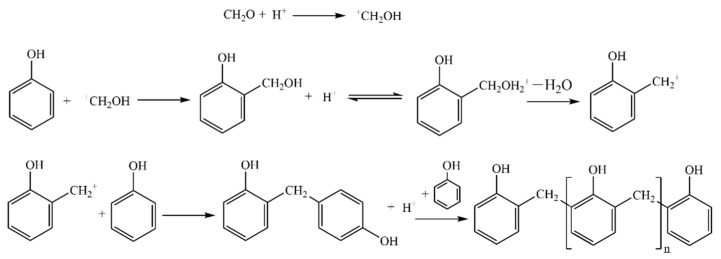
Synthesis process and structure of conventional phenolic resins without modification.

In addition, it should be mentioned that unmodified phenolic resin (UMPR) with a 0% MTMS addition is the phenolic resin commonly utilized in the current rail grinding wheel production industry. It is a linear or slightly branched polymer, and its conventional synthesis and structure are displayed in Figure 14. The specific steps of synthesis are as follows: In the presence of an acidic catalyst, formaldehyde becomes more electrophilic, and hydrogen ions combine to produce carbon cations. The hydroxymethyl (-CH_2_OH) is then substituted with the phenolic hydroxyl group in phenol at the adjacent and opposite positions to form hydrogen ions and bi-phenolic methane. Eventually, as the polycondensation reaction proceeds, macromolecular-chain phenolic resin is produced [40].

### 3.2. Preparation and Curing of Silicone-Modified Phenolic Resin Molded Specimens

The mold size for the preparation of resin specimens was 8 mm × 8 mm × 100 mm. A mixture of willow wood powder and calcium carbonate was selected as the filler for the resin-molded specimens, and the mixture was mixed evenly with SMPR in the ratio of m (resin powder): m (willow wood powder): m (calcium carbonate) as 5:3:2.

The preparation process of the resin specimens was as follows. The molds were firstly preheated at 180 °C for 5 min in the flat vulcanizer, and then the mixture of the prepared resin specimens was pressed into the molds at 180 °C for 20 min. After that, the molds were cooled to room temperature, and the resin specimens were demoulded. Finally, the prepared resin specimens were subjected to secondary curing at 80 °C, 100 °C, 120 °C, 140 °C and 160 °C for 2 h and at 180 °C for 4 h, which completed the preparation and curing of the SMPR molded specimens.

### 3.3. Materials and Characterization

The following reagent materials were used in this study: phenol (analytical purity, Kemiou Chemical Reagent Co., Ltd., Tianjin, China), methyl-trimethoxy-silane (MTMS, analytical purity, Aladdin, Shanghai, China), paraformaldehyde (POM, analytical purity, Guangfu Fine Chemical Research Institute, Tianjin, China), hexamethylenetetetramine (HMTA, analytical purity, Yihua Tongbiao Technology, Beijing, China), p-toluenesulfonic acid (TsOH, analytical purity, Macklin Biochemical Co., Ltd., Shanghai, China).

The following instruments were used in the study for characterization: Fourier Transform Infrared Spectrometer (IRPrestige-21, Shimadzu Instruments, Kyoto, Japan), Differential Scanning Calorimeter (DSC200F3, NETZSCH, Bavarian Free State, Germany), Electronic universal testing machine (WDW-50, Yongke, Jinan, China), Impact strength testing machine (TY-4021A, Tianyuan, Suzhou, China), Thermogravimetric Analyzer (STA409C, NETZSCH, Bavaria, Germany), Scanning Electron Microscope (Inspect F50, FEI, Hillsboro, OR, USA).

Analysis of infrared spectra is a common method for detecting the structure and group species of polymers, which can be used to determine the composition of the macromolecular chain structure and alterations in the branching groups of modified phenolic resins [41]. In this examination, the infrared spectroscopy test scanning range was 4000–400 cm^−1^, and the number of scans was 32. The test method was the KBr pressed-disk technique. Before preparing the sample, the resin and KBr powder were dried for 12 h in a vacuum oven to remove the effect of excess moisture. Resin and KBr powder were subsequently mixed into powder and pressed into 0.5 mm flakes for testing. The resin specimens for the fluidity test were a circular sheet with a thickness of 4.80 ± 0.20 mm and a diameter of 12.50 ± 0.30 mm. On a platform with an inclination angle of 60 degrees and a roughness of 5.29 nm, the fluidity of the specimens was measured by examining their flow distance within 20 min. The flow distance of the resin is measured using a digital vernier caliper. The temperature of the test was 125 ± 1 °C. The fluidity of phenolic resin prepared for rail grinding wheels is generally between 30 mm and 60 mm [42]. Differential scanning calorimetry (DSC) was used to measure the curing temperature of resin specimens. The dried resin specimens were equilibrated at 30 °C for 2 min under the protection of N_2_ gas with a flow rate of 20 mL/min before being heated to 220 °C at a rate of 10 °C/min. The heat change during the process was recorded to analyze the curing temperature of the resin. The bending strength of resin specimens was measured by a three-point bending test, according to ASTM D 7264/D 7264M-2007 [43]. The impact strength of resin specimens was measured by a non-instrumented impact test, according to ISO 179-1-2010 [44]. All experiments were conducted under ambient conditions (temperature: 15–21 °C; humidity, 40–60%).

## 4. Conclusions

In order to improve the mechanical strength and grinding performance of rail grinding wheels by optimizing the thermal stability and mechanical properties of the phenolic resin binder, silicone-modified phenolic resin (SMPR) with excellent thermal resistance was prepared by using MTMS as the organosilicone modifier in industrially applicable transesterification and polymerization reactions. Additionally investigated was the effect of organosilicon modifier MTMS concentration on the performance of SMPR. The primary resulting conclusions are as follows:

1. The infrared spectroscopy tests showed that the silicone-modified phenolic resin (SMPR) was synthesized by successively introducing Si-O and Si-C bonds with greater bonding energy in MTMS into the macromolecular chain structure of phenolic resin through transesterification reactions and copolymerization reactions under the catalysis of p-toluenesulfonic acid;
2. The fluidity of silicone-modified phenolic resin (SMPR) was between 30 and 60 mm, exhibiting excellent processability;
3. Differential scanning calorimeter results indicated that silicone-modified phenolic resin (SMPR) can be fully cured at temperatures greater than 160 °C and that the SMPR has a lower curing temperature than the unmodified conventional phenolic resin (UMPR);
4. Thermogravimetric analysis revealed that the thermal stability performance of the MTMS-modified SMPR was superior to that of the UMPR. When the amount of MTMS was 40% of the phenol mass, the thermal weight loss temperature of the synthesized SMPR at 30% weight loss was approximately 66% higher than that of the UMPR, which exhibited the best thermal stability;
5. According to the results of mechanical property testing, the bending strength and impact strength of SMPR modified with 40% MTMS were approximately 14% and 6% greater than those of UMPR, suggesting the best mechanical performance;
6. The grinding test findings demonstrated that the grinding ratio of rail grinding wheels prepared with 40% MTMS-modified SMPR as a binder was approximately 39.6% higher than that of rail grinding wheels manufactured with UMPR, extending the service life of rail grinding wheels.

## Figures and Tables

**Table 1:** Specific formulas of SMPR with different MTMS content *.

Sample	Mass/g			
	Phenol	MTMS	TsOH	POM
UMPR (MTMS-0%)	94.11	0	0.94	24.02
SMPR (MTMS-10%)	94.11	9.41	0.94	24.02
SMPR (MTMS-20%)	94.11	18.82	0.94	24.02
SMPR (MTMS-30%)	94.11	28.23	0.94	24.02
SMPR (MTMS-40%)	94.11	37.64	0.94	24.02
SMPR (MTMS-50%)	94.11	47.06	0.94	24.02

* The above reaction ratios between phenol and POM are molar ratios, n (phenol): n (POM) = 1:0.8, and the reaction ratio between phenol, MTMS, and BA are mass ratio, m (phenol): m (MTMS) = 1: (0.1~0.5), m (phenol): m (TsOH) = 1:0.

## Data Availability

The datasets used or analyzed during the current study are available from the corresponding author on reasonable request.

## Sample Availability

Not available.
